# Diversity Analysis and Comprehensive Evaluation of 101 Soybean (*Glycine max* L.) Germplasms Based on Sprout Quality Characteristics

**DOI:** 10.3390/foods13213524

**Published:** 2024-11-04

**Authors:** Xiaoyan Zhang, Gufeng Wu, Yuhe Wu, Ning Tang, Lu Huang, Dongqing Dai, Xingxing Yuan, Chenchen Xue, Xin Chen

**Affiliations:** 1 Institute of Industrial Crops, Jiangsu Academy of Agricultural Sciences, Nanjing 210014, China; xyzhang@jaas.ac.cn (X.Z.); wgf991103@163.com (G.W.); huanglu@jaas.ac.cn (L.H.); daidongqing13@126.com (D.D.); yxx@jaas.ac.cn (X.Y.); cx@jaas.ac.cn (X.C.); 2 School of Food and Biological Engineering, Jiangsu University, Zhenjiang 212013, China; 3 School of Food Science, Nanjing Xiaozhuang University, Nanjing 211171, China; 17705249959@163.com (Y.W.); tangning@njxzc.edu.cn (N.T.)

**Keywords:** soybean sprouts, quality characteristics, parameter screening, principal component analysis (PCA), comprehensive evaluation

## Abstract

Soybean sprouts are a common culinary vegetable due to their high nutrition and tasty flavors. To select soybean varieties with excellent sprout quality, 101 soybean materials were collected from different regions of China, and eight sprout quality parameters were determined for overall evaluation. The results showed that eight sprout quality parameters achieved varying degrees of difference and correlation. Based on the principal component analysis (PCA), three principal components were extracted, with a cumulative contribution rate of 78.314%. Further, the comprehensive evaluation value (D) of soybean sprout quality was calculated by membership function analysis based on PCA, and the quality of soybean sprouts was ranked accordingly. Subsequently, a regression equation for the prediction of soybean sprout quality was established using a stepwise regression analysis, and the model showed a good prediction performance (correlation coefficient of prediction > 0.8; residual predictive deviation > 2.0). On these grounds, it was proposed that the quality of soybean sprouts could be comprehensively predicted by four parameters: hypocotyl length, edible rate, 100-seed weight, and total isoflavone content and saponin content. In conclusion, this study provides excellent varieties for soybean sprout production and new variety breeding, and it provides an important reference for the prediction of soybean sprout quality.

## 1. Introduction

The soybean (*Glycine max* L.) is an important food and cash crop worldwide. Soybeans are a rich source of protein for humans and animals, and the nutrient composition of soybeans, including lipids, carbohydrates, and functional metabolites, comprises indispensable parts of a healthy diet [1,2]. The edible soybean sprouts from germinated soybeans can be produced within 5–7 days. Soybean sprouts can be easily grown by both specialized producers and consumers, and their production is not affected by seasonal restrictions. In Asian countries, soybean sprouts are considered traditional dietary vegetables year-round. For example, more than 500,000 tons of soybean sprouts are consumed annually in Korea [3]. There is a growing recognition of the health benefits of including soybean sprouts in the daily diet, and higher consumption of soybean sprouts is associated with a lower incidence of cardiovascular diseases and cancer [4]. Consumption of soybean sprouts has increased in Europe and the United States in recent decades [1]. Therefore, producing high-quality soybean sprouts is crucial, especially in winter or in areas with extreme climates, where vegetable supply is limited.

Germination is a straightforward and effective way to improve the nutritional value and reduce the anti-nutritional factors in soybeans [4,5]. There is growing evidence showing that soybean sprouts possess higher nutritional value than their seed counterparts [6,7]. Generally, the content and composition of nutrients in soybean seeds undergo significant changes during germination. On one hand, macro-molecules such as proteins, polysaccharides, and lipids may break down into free amino acids, monosaccharides, and fatty acids, respectively, after germination. On the other hand, the content of bioactive components increases after germination, which considerably improves the nutritional value of soybean sprouts. For instance, there are studies where the content of isoflavones and vitamin C in soybean sprouts was found to be substantially higher than in the seeds [8]. In recent years, soybean sprouts have garnered increased attention as functional foods due to their rich content of bioactive components including but not limited to phenolic compounds. The health benefits of dietary phenolic compounds have recently drawn attention primarily due to their potent antioxidant properties and their role in preventing oxidative stress-related diseases such as cancer [9]. Isoflavones, a class of phenolic bioactive compounds structurally similar to mammalian estrogens, are well-known antioxidants and phytoestrogens [10]. There is evidence to suggest that phenolic compounds are one of the major bioactive components abundantly present in soybean sprouts [7,11,12,13].

To produce high-quality soybean sprouts, it is essential to consider several characteristics, including their growth parameters and nutrition content. The development of these traits during sprouting is closely linked to the genotypes of soybeans. Therefore, identifying a suitable soybean variety for sprouting is of great significance. China, where soybeans were first cultivated approximately 3000 years ago, boasts a wealth of diverse genetic resources that significantly aid in the selection of suitable sprouting varieties and the enhancement of soybean genetics. In recent years, efforts had been directed towards selecting suitable soybean varieties through a comprehensive assessment of the target traits of soybean germplasm resources. For instance, 175 soybean varieties were evaluated for genotypic variability in the functional phytochemicals (lutein, tocopherols, and phytosterol), which provided a valuable foundation for soybean breeding [14]. Additionally, the fatty acid composition of 1025 soybean accessions from different ecoregions were evaluated, revealing distinct compositions among the regions [15]. To the best of our knowledge, however, there have been no comprehensive studies on the evaluation of soybean sprout characteristics.

In this study, the growth parameters and the bioactive components of soybean sprouts were determined using 101 soybean varieties collected from different regions of Jiangsu Province, one of the largest soybean sprout-consumption provinces in China. This study aims to provide a foundational understanding for selecting promising soybean varieties suitable for sprout production. Additionally, this study seeks to establish a preliminary evaluation system for the quality traits of soybean sprouts, providing a valuable reference for the reliable varietal selection in soybean germplasm resources for sprouting and for breeding programs.

## 2. Materials and Methods

### 2.1. Plant Materials and Growth Conditions

The 101 varieties of soybean were collected by the Institute of Industrial Crops, Jiangsu Academy of Agricultural Sciences (Nanjing, China). The variety name, sample number, and source information are shown in Table S1. Soybean seeds were stored at −4 °C until use.

The mature and undamaged soybean seeds were selected for sprouting. Ten grams of soybean seeds were sterilized with 1% (*v*/*v*) sodium hypochlorite for 15 min and then washed three times with distilled water. The surface disinfection procedure can effectively kill the microorganisms on the seed surface and prevent the soybean sprouts from decaying during germination. Subsequently, soybean seeds were soaked in distilled water for 8 h at room temperature. The soaked seeds were spread evenly on the seedling tray of a growth chamber and cultured in the dark at 25 °C and with an average humidity of 75%. The germinating soybean seeds were sprayed with water every 4 h. Soybean sprouts were harvested 5 days after germination. The growth parameters were measured at harvest time. The shoots of soybean sprouts were regarded as the edible sprout part in this study. The edible parts of at least 10 different soybean sprouts were frozen in liquid nitrogen immediately after harvesting and subsequently lyophilized in a freeze dryer (Scientz-12N/C, Xinzhi, Ningbo, China). The lyophilized samples were grounded into fine powder and stored at 4 °C for subsequent determination of nutritional components content.

### 2.2. Measurement of Growth Parameters

Fifteen representative sprouts seedlings were selected as the samples for each soybean variety. The hypocotyl length and diameter were measured using a ruler and micrometer caliper, respectively. For the measurement of total fresh weight and edible fresh weight, five sprouts were set as a sampling group, and three biological replicates were used. The edible rate was calculated as in Equation (1):

(1) $$\mathrm{Edible}\;\mathrm{rate}\left(\%\right)=\frac{\mathrm{deible}\;\mathrm{fresh}\;\mathrm{weight}}{\mathrm{total}\;\mathrm{fresh}\;\mathrm{weight}}\times 100\%$$

### 2.3. Determination of Total Phenolic Content

A one gram sample of lyophilized sprouts was extracted with 10 mL of 70% (*v*/*v*) methanol at room temperature. After centrifugation (12,000 rpm, 4 °C, 20 min), the supernatant was collected as the extraction solution for subsequent use. Total phenolic content (TPC) was determined according to the Folin–Ciocalteu assay, as reported in the study of Tian [16]. Gallic acid was used as a standard, and the results are expressed as mg of garlic acid equivalents (GAE) per g dry weight (DW). All the spectrophotometric measurements were performed using a microplate reader (Spark 10M, TECAN, Männedorf, Switzerland), on the basis of the literature [17].

### 2.4. Determination of Isoflavone Content

The total isoflavone content (TIC) was determined according to the previous methods of Dwiatmaka et al. [18] and Migues et al. [19], with some modifications. Briefly, 1 g soybean sprout powder was extracted with absolute ethanol under an ultrasonic bath at room temperature for 1 h. The extraction was repeated twice, and the supernatant was collected and vacuum-dried (Concentrator plus, Eppendorf, Hamburg, Germany). The residue was dissolved in the extraction solution, and then, 100 μL of extracts were diluted 100 times to produce the test solution. The total isoflavone content was determined spectrophotometrically at 260 nm in a quartz 96-well microplate. Genistein was used as the standard. The results are expressed as μg of genistein equivalents (GE) per g DW.

### 2.5. Determination of Saponin Content

The saponin content was determined according to the method of Chen et al. [13], with some modifications. The lyophilized sprout samples (0.5 g) were extracted with 10 mL of 80% methanol (*v*/*v*) at room temperature and then centrifuged (12,000 rpm, 4 °C, 20 min). The supernatant was collected for saponin determination. Next, 0.1 mL of supernatant, 0.4 mL of 80% methanol (*v*/*v*), 0.5 mL of 8% vanillin solution (*m*/*v*), and 5 mL of 72% H_2_SO_4_ were well mixed in an ice-water bath. The absorbance of the mixture was measured spectrophotometrically using a 96-well microplate at 544 nm. The content of saponin was calculated based on the standard curve and is expressed as mg g^−1^ DW.

### 2.6. Determination of γ-Aminobutyric Acid (GABA) Content

To quantify the content of GABA, 0.5 g of lyophilized sprout powder was homogenized in 5 mL of 70% (*v*/*v*) ethanol at room temperature for 1 h. After centrifugation (8000 rpm, 4 °C, 5 min), the supernatant was collected. The extraction was repeated twice, and the supernatant was combined and evaporated using a vacuum concentrator (Concentrator plus, Eppendorf, Germany). In the tested tube, 0.1 mL of concentrated extract was mixed with a mixed reaction solution containing 0.2 mL of 0.2 M borate buffer (pH 9.0) and 1.0 mL of 6% phenol reagent (*v*/*v*); then, 0.4 mL of 7.5% sodium hypochlorite (*m*/*v*) was added. The absorbance of the reaction mixture was determined at 630 nm according to the methods of Sharma et al. [20]. The GABA content was determined from a calibration curve prepared using the standard GABA.

### 2.7. Statistical Analysis

The statistically analyzed data are presented as the mean ± standard error (SE) after one-way analysis of variance (ANOVA) by SPASS 26.0 (IBM, Armonk, NY, USA). The Duncan test was used to evaluate significant differences among the data at *p* < 0.05. The sprout characteristics of soybean sprouts were comprehensively evaluated by PCA and fuzzy mathematics. PCA scores scatter plot and PCA loading plot were created by OriginPro 2021 (Originlab Corporation, Northampton, MA, USA). The membership function analysis was preformed according to the methods previously reported [21,22]. When calculating the membership function value (*U*) of the quality characteristics of soybean sprouts, Equation (2) was used to standardize the primitive data. In Equation (2), *X_i_* represents the *i*th principal component value of soybean sprouts; *X_i, max_* and *X_i, min_* represent the maximum and minimum values of the *i*th principal components, respectively. The weight coefficient (*w*) was calculated by Equation (3), in which *p_i_* represents the contribution rate of the *i*th principal component. Finally, the comprehensive evaluation value (*D*) was determined by Equation (4).

(2) $$U(X_{i})=(X_{i}-X_{i,\;min})/(X_{\;i,\;max}-X_{\;i,\;min}),i=1,2,\ldots n$$

(3) $$w_{i}=p_{i}/\sum_{i=1}^{3}p_{i},\;\;i=1,2,3$$

(4) $$D=\sum_{i=1}^{n}{U(X_{i})w}_{i},\;i=1,2,\ldots n$$

## 3. Results

### 3.1. Variation on the Quality Characteristics of Soybean Sprouts

**Figure 1:**
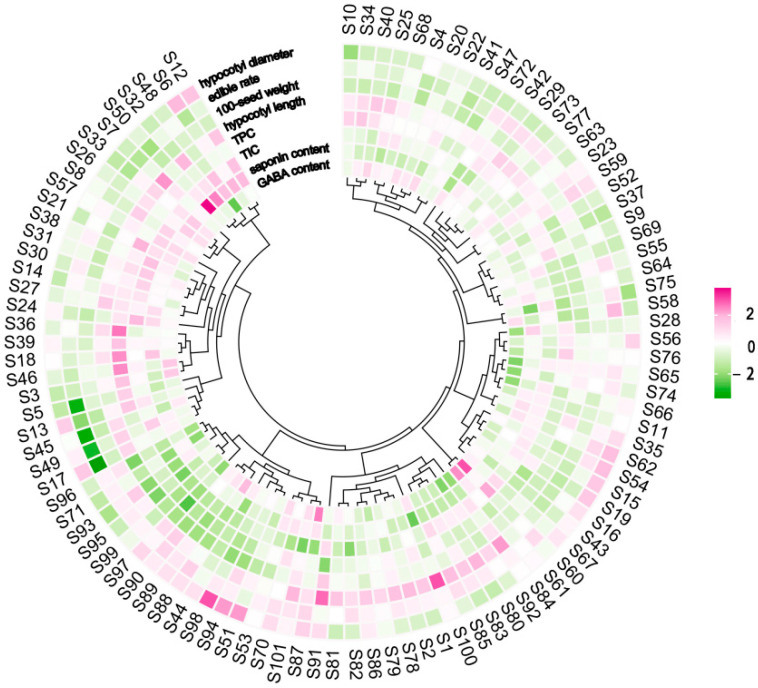
The heat map of the quality characteristics of 101 soybean sprouts.

The variability of the quality characteristics of 101 soybean sprouts was largely different, reflecting a high genetic diversity of soybean germplasm resources (Figure 1). Generally, the distribution of the eight sprout quality characteristics fitted a normal distribution (Figure S1). Specifically, the hypocotyl length of soybean sprouts was mainly distributed between 7.00 and 10.00 cm (Figure S1A), the hypocotyl diameter was mainly distributed between 1.70 and 3.00 mm (Figure S1B), the edible rate was between 85.00 and 95.00% (Figure S1C), and the 100-seed weight was between approximately 15.00 and 25.00 g (Figure S1D). Additionally, TPC and TIC were mainly distributed between 4.00 and 7.00 mg GAE g^−1^ DW and 7.75 and 8.50 μg GE g^−1^ DW (Figure S1E,F), respectively. The saponin and GABA content were mainly distributed between 40.00 and 80.00 mg g^−1^ DW and 3.00 and 6.00 mg g^−1^ DW, respectively (Figure S1G,H).

The coefficient of variation (CV) of the edible rate was 4.49% (Table 1). In contrast, the CVs for the other seven sprout characteristics were all greater than 10.00%, with the maximum CV observed in 100-seed weight (41.79%). The maximum value of 100-seed weight was 5.45 times of the minimum value at 26.54. The CV of hypocotyl length was 37.10%, with the maximum value of germination rate being 8.90 times the minimum value at 12.80. The CV of hypocotyl diameter was 12.00% at 1.30. Regarding the bioactive components content, the CVs for TPC, TIC, and saponin content were approximately 20.00%. The CV of GABA content was 38.73% at 8.82. The above results indicate that there were significant differences in the quality characteristics of soybean sprouts, which could be further used for the comprehensive evaluation of sprout quality.

### 3.2. Correlation Analysis

**Figure 2:**
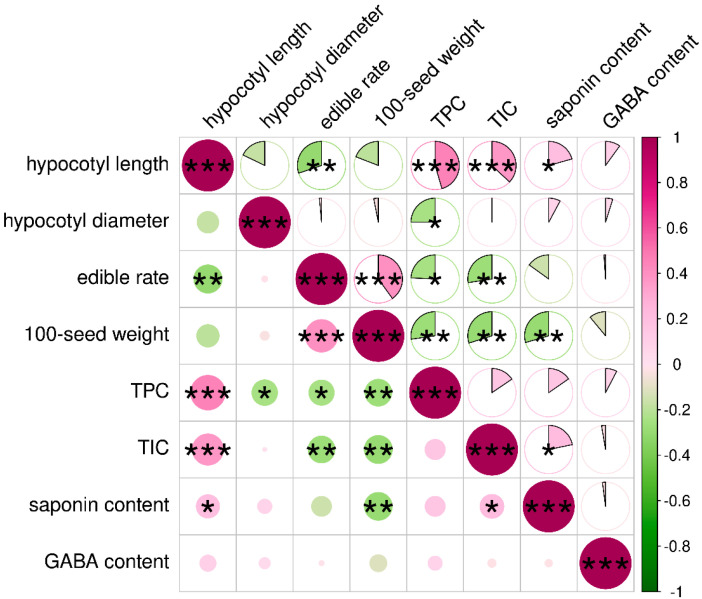
Correlation analysis of quality characteristics of soybean sprouts. The size of the circle represents the value of the Pearson coefficient, and red indicates a positive correlation, green indicates a negative correlation. * means the significant is at the level of *p* < 0.05; ** means the significant is at the level of *p* < 0.01; *** means the significant is at the level of *p* < 0.001.

The correlation analyses of sprout quality characteristics revealed several significant relationships (Figure 2). Specifically, there were highly significant positive correlations between hypocotyl length and TPC as well as hypocotyl length and TIC (*p* < 0.001), and a significant positive correlation was found between hypocotyl length and saponin content (*p* < 0.05). Additionally, a highly significant positive correlation was found between edible rate and 100-seed weight (*p* < 0.001). Significant positive correlations were also observed between TIC and saponin content (*p* < 0.05). Conversely, a strong significant negative correlation was found between hypocotyl length and edible rate (*p* < 0.01). Furthermore, significant negative correlations were observed between hypocotyl diameter and TPC as well as edible rate and TPC (*p* < 0.05), and a strong significant negative correlation was found between edible rate and TIC (*p* < 0.01). The 100-seed weight exhibited strong negative correlations with TPC, TIC, and saponin (*p* < 0.01).

### 3.3. The PCA Analysis of Quality Characteristics Parameters of Soybean Sprouts

To minimize the data dimensionality, the eight quality characteristics parameters of 101 soybean sprouts were further analyzed by subjecting to PCA. According to the Kaiser’s criterion of eigenvalues greater than 1, three principal components were extracted with variance contribution rates of 33.600%, 26.934%, and 17.780%, respectively (Table 2). The first principal component (PC1), with the highest variance contribution rate of 33.600%, was identified as the most significant, representing 33.600% of the original information regarding soybean sprouts’ quality characteristics. The variance contribution rate of the second principal component (PC2) and the third principal component (PC3) decreased in turn, indicating a decreasing level of importance. The cumulative contribution rate of these three principal components reached 78.314%, demonstrating their ability to explain 78.314% of the original information in the variables.

**Figure 3:**
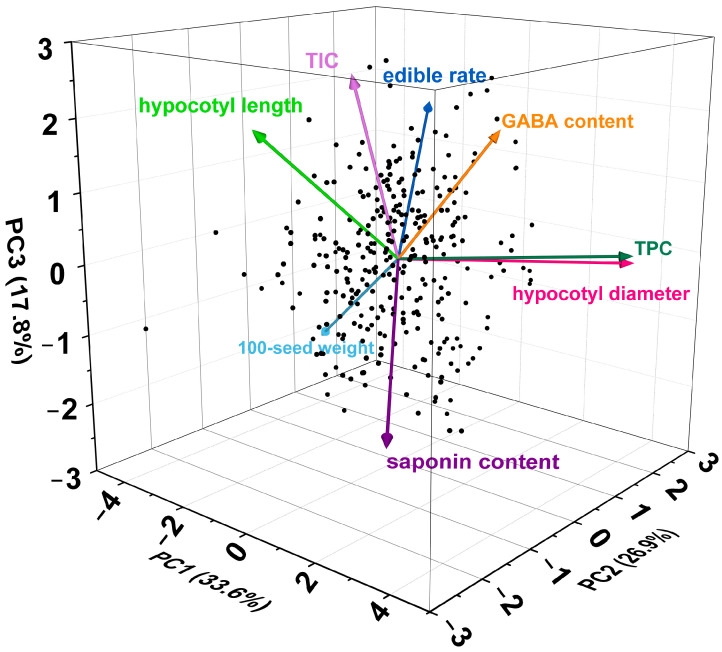
The PCA plot of the quality characteristics of 101 soybean sprouts.

The loading matrix of the three principal components on each of the characteristic parameters is presented in Table 2. The higher the absolute value of the loadings, the greater their contribution to the principal components. Based on this, the highest absolute load value of PC1 was observed in TPC, 100-seed weight, and hypocotyl diameter, indicating that PC1 was closely associated with these three parameters. Similarly, PC2 was mainly related to edible rate and saponin content. PC3 was predominantly associated with TIC and hypocotyl length (Table 2, Figure 3). Therefore, three independent comprehensive indices were identified through PCA, which could be employed to replace the original variables for a comprehensive evaluation of the quality of soybean sprouts.

### 3.4. Comprehensive Evaluation of Sprout Quality Characteristics of 101 Soybean Varieties Based on PCA

Using the principal component values of the sprout quality characteristics indexes for each soybean material, the corresponding membership function values were calculated according to Equation (2) (Table S2). The results indicated that the membership function values for each soybean sprout variety were distinct. For PC1, the highest U(X1) value (5.471) was observed in S50 soybean sprouts, suggesting that this variety of soybean sprout exhibited the best quality on the composite index of PC1. Conversely, the lowest U(X1) value (−1.000) was observed in S100 soybean sprouts, indicating that it exhibited the worst quality on the index of PC1. Similarly, the maximum (3.784) and minimum (−1.000) values of U(X2) were observed in S95 and S49 soybean sprouts, respectively, which suggests that S95 and S49 soybean sprouts exhibited the best and worst quality performance on the composite indicator of PC2, respectively. For U(X3), S25 and S93 showed the best and worst performers on the composite index PC3, respectively.

Based on the contribution rates of each comprehensive index (Table 2), the weight coefficients for the three comprehensive indexes were calculated with Equation (3), yielding values of 0.429, 0.344, and 0.227, respectively. Subsequently, the *D* value, representing the comprehensive evaluation value of soybean sprout quality characteristics, was calculated using Equation (4), and the quality of the soybean sprouts was ranked according to this value (Table S3). The analysis revealed that the ten varieties with the highest quality ranking for soybean sprouts, indicating their suitability for producing soybean sprouts, were S44, S4, S75, S99, S95, S98, S88, S43, S58, and S19 in order. Conversely, the ten varieties with the lowest quality ranking, suggesting a lesser suitability for producing soybean sprouts, were S91, S84, S100, S101, S79, S78, S83, and S101, S79, S78, S83, S2, S80, and S3, in descending order.

### 3.5. The Establishment of a Regression Model and Quantitative Model for Quality Characteristics of Soybean Sprouts

**Figure 4:**
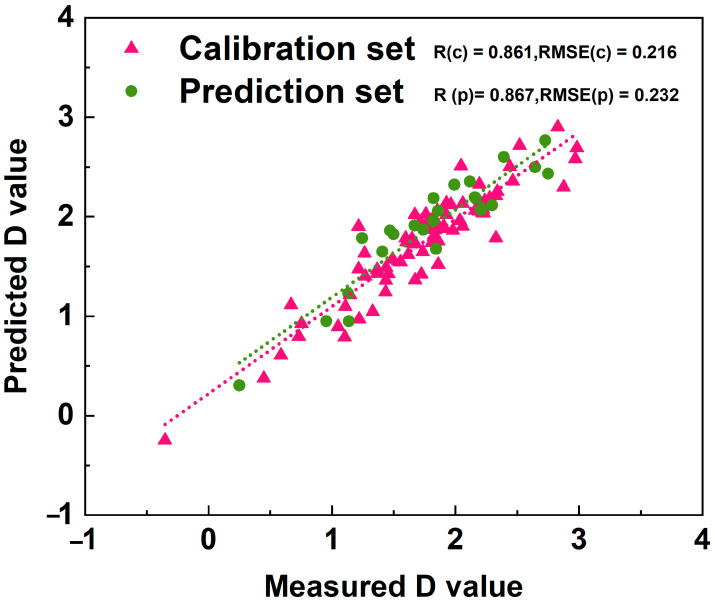
The scatter plots of the calibration and prediction *D* value of quality characteristics of soybean sprouts. R(c) and R(p) represent the correlation coefficient of the calibration and prediction set, respectively. RMSE(c) and RMSE(p) represent the correlation coefficient of the calibration and prediction set, respectively.

To identify suitable indicators for the characterization of soybean sprout quality, the 101 soybean materials were randomly partitioned into a calibration set and a prediction set in a ratio of 4:1. Out of these, 76 materials (about 3/4 of the total) were randomly selected to develop regression equations using stepwise regression analysis. The remaining 25 materials (about 1/4 of the total) were used to validate the reliability of the model. In the regression analysis, which considered the comprehensive evaluation values of sprout quality characteristics as the dependent variables and the membership function value of each characteristic index as independent variables, the optimal regression equation was as follows:

*D* = 3.606 − 0.263*X*_1_ + 0.106*X*_3_ − 0.566*X*_4_ − 0.082*X*_6_ − 0.115*X*_7_ (*R*^2^ = 0.951, *p* < 0.01)

where *D* is the membership functions’ value of sprouts quality for one cultivar, and *X*_1_, *X*_3_, *X*_4_, *X*_6_, and *X*_7_ represent the membership function value for hypocotyl length, edible rate, 100-grain weight, and total isoflavone content and saponin content, respectively. The coefficient of determination *R*^2^ and *p*-value of the equation indicate that the equation could effectively evaluated the integrated assessment of sprout quality characteristics across different soybean varieties (Figure 4). Moreover, the accuracy of the model was assessed. The root mean square error (RMSE) value of the calibration and the prediction set was 0.216 and 0.232, the correlation coefficient was 0.861 and 0.867, and the relative percentage difference (RPD) was 2.682 and 2.742, respectively (Figure 4). The above results indicate that the established model is highly reliable and exhibits excellent predictive capabilities.

## 4. Discussion

Soybean sprouts, a common vegetable in Asian cuisine, are favored for their nutritional value, crunchy texture, and pleasing taste. Their cultivation process is straightforward, enabling year-round production and consistent supply. The nutritional value of soybean sprouts is higher than that of their seeds counterparts [6]. The sprouting process consumes large molecules of proteins and fats stored in the seeds, making soybean sprouts lower in calories [7]. Furthermore, the germination of soybeans significantly increases the levels of certain bioactive substances, making them a functional food in line with modern health standards [23,24]. In the production of soybean sprouts, varieties with high germination rates are typically chosen, and their production has mainly focused on yield and appearance quality. However, the intrinsic nutritional quality of soybean sprouts has been largely overlooked. In addition, previous studies on the nutritional quality of soybean sprouts primarily focused on the effects of exogenous additives (e.g., Ca^2+^) and exogenous stimuli (e.g., light environment and temperature) on the nutritional compounds content of soybean sprouts [25,26]. There is a scarcity of studies on the comprehensive evaluation of the post-sprouting quality characteristics of different soybean varieties, and there are no reported systems for evaluating soybean sprout characteristics. Evaluating the sprout characteristics of soybean germplasm resources is crucial for identifying materials with excellent traits for sprout production and for breeding soybean varieties optimized for sprouts.

It is very important to select the appropriate parameters for identifying the characteristics of soybean sprouts. Previous studies have identified the quality characteristics of soybean sprouts, including growth and nutritional quality parameters [25,27,28,29]. Several studies have investigated the differences in sprout quality-related indexes among varieties using collected soybean germplasm resources. For example, Dhakal et al. [30] collected 12 soybean materials and studied the differences in unsaturated fatty acid. Bi et al. [31] investigated the changes in flavonoids during the germination of two soybean varieties using metabolomics techniques. It is noteworthy that the quality of soybean sprouts is determined by both growth and nutritional parameters, which are jointly influenced by genotype and environment, with the genotype playing a predominant role [32]. The limited number of soybean materials studied in the previous research resulted in a narrow genetic base. In this study, 101 soybeans germplasm resources collected from various regions of Jiangsu Province, China, were used as test materials, providing a rich genetic diversity with abundant variation in sprout characteristics (Figure 1, Table S1), which can be beneficial for screening excellent soybean varieties for sprouts. On this basis, it is expected to better screen appropriate parameters for evaluating the quality characteristics of soybean sprouts.

It has been documented that soybean varieties suitable for soybean sprout production should meet some criteria, including a high germination rate (≥90%), small seed size (4–15 g/100 g seeds), good sprout length (8–12 cm), and thick hypocotyl (2.0–2.2 mm), etc. [27]. The seed vigor of soybeans is particularly sensitive to external influences and prone to decline during storage [33]. The preference for small-grain soybeans in sprout production is due to their higher seed vigor and germination rate, which is crucial for ensuring a good yield. d. The 100-seed weight ranged from 5.97 g to 32.51 g (Figure 1 and Figure S1), with the top 10 selected soybean materials having a 100-seed weight ranging from 4 g to 15 g (Table 1, Figure S1). This indicates that the soybean materials screened according to the methodology of this study basically meet the needs for sprout production in terms of the 100-seed weight. Phenolic compounds are one of the most prominent secondary metabolites in plants, exhibiting antioxidant and anti-inflammatory properties [34]. The abundance of phenolics in soybean sprouts makes them an indispensable part of a healthy diet. Previous research has noted considerable variability in the TPC among different soybean accessions. For example, the TPC ranged from 13.35 to 21.49 mg GAE g^−1^ DW, and the TPC was higher in black-seedcoat soybeans than in yellow-seedcoat soybeans [35]. The present study showed that the content of TPC in 101 soybean sprouts ranged from 0.99 to 4.57 mg GAE g^−1^ DW, and a large variation of TPC among the studied materials was observed (Table 1). It was reported that the TPC of the two varieties of soybean sprouts was about 4 mg GAE g^−1^ DW with no significant difference, which could possibly be attributed to the close genetic relationship of the two studied soybean materials [25]. Isoflavone, a type of phytoestrogen, is a highly representative bioactive substance in soybeans. It was noticed that the isoflavone content of soybean sprouts of different genotypes varied greatly (Table 1, Figure 1). These results are consistent with the findings on the isoflavone profile in different soybean sprouts [36]. In recent years, GABA in soybean and soy-based products has garnered attention for its physiological activities, such as antioxidant benefits, immunity regulation, and improvement of the nervous system [37]. However, there is a lack of research analyzing and comparing the content of saponins and GABA in soybean germplasm resources. The variation in sprout quality characteristics among different genotypes of soybean, as indicated by the growth indicators and the results on bioactive compounds, underscores the impact of genetic diversity on sprout quality in soybeans.

To select appropriate indicators to evaluate the sprout quality characteristics, a correlation analysis was carried out (Figure 2). The highly significant positive correlation (*p* < 0.001) between edible rate and 100-seed weight could be attributed to the fact that soybeans with a higher 100-seed weight have larger cotyledons, which are one of the main edible parts of soybean sprouts. The role of GABA in soybean resistance to stress and in regulating the accumulation of phenolic compounds in soybean sprouts has been documented [37,38]. In this study, there was a significant positive correlation (*p* < 0.05) between TIC and saponin content. It was reported that small- and medium-sized soybean seeds have high levels of isoflavones [39]. Additionally, small soybean seeds had the highest TPC, and large soybean seeds had the lowest TPC [40]. Similar results were found in this study, as there was a strong significant negative correlation (*p* < 0.01) between edible rate and TIC and between 100-seed weight and TPC (Figure 2). These findings indicate that there was information redundancy among the eight indicators, which hindered their effectiveness in evaluating soybean sprout quality. Consequently, these indicators require refinement to enhance the efficiency and accuracy of soybean sprout quality evaluation.

Previous studies have shown that multivariate statistical methods such as PCA and membership function analysis have been extensively utilized to analyze the quality differences of agricultural products and the stress resistance of crops, including cocoa beans, potatoes, peanut, and cabbage [22,41,42,43]. In this study, this method was employed, and the eight sprout quality characteristics were first transformed into three principal component factors through PCA, which collectively accounted for 78.314% of the variation. Subsequently, the three independent comprehensive indexes derived from PCA were selected to replace the original eight quality characteristics indicators for the analysis and evaluation of sprouting characteristics. PC1 mainly integrated the TPC, 100-seed weight, and hypocotyl diameter of soybean sprouts. The top three varieties with the highest scores in PC1 were S50, S32, and S48, indicating that they excelled compared to other varieties in terms of TPC, 100-seed weight, and hypocotyl diameter. Similarly, S95, S44, and S75 demonstrated superior performance in edible rate and saponin content (PC2), while S25, S75, and S4 excelled in TIC and hypocotyl length (PC3) (Figure 3). Furthermore, to identify the varieties with outstanding performance across the three comprehensive indicators, the comprehensive evaluation *D* values of different soybean sprouts were calculated using the subordinative function value. Then, the quality characteristics of soybean sprouts were ranked based on the magnitude of the *D* values. Consequently, the top 10 soybean materials most suitable for sprouts production were identified as S44, S4, S75, S99, S95, S98, S88, S43, S58, and S19, in descending order of *D* value (Table S3).

To ensure the accuracy and objectivity of the comprehensive evaluation, a simple and reliable evaluation method is required. In this study, a mathematical model for the evaluation of the quality characteristics of soybean sprouts was established by using stepwise regression analysis (Figure 4). It was proposed that the quality characteristics of soybean sprouts can be evaluated based on five indicators: hypocotyl length, edible rate, 100-seed weight, and total isoflavones and saponin content. The higher correlation coefficient and RPD values and lower RMSE values were considered to reflect a better model performance [44]. Therefore, the sprout quality characteristics of soybeans can be evaluated according to the value of *D*. The above results have important reference value for the rapid evaluation of soybean sprout characteristics. It is worth noting that the differences in quality characteristics among different soybean varieties are not only influenced by genotypes but are also closely related to the climate origin of the soybean. In addition to the parameters determined in this study, other parameters, such as fatty acids content, protein content, amino acids, and trace elements content, also have different effects on sprout quality. Therefore, the model established here has its limitations. To enhance the accuracy of the model and broaden its application, further studies should collect a broader range of soybean germplasm resources (preferably from multiple years and locations) and determine more quality parameters, thereby adding more data to the evaluation model. Based on this, a high-precision evaluation model of soybean sprout characteristics with a wide application range can be finally developed.

## 5. Conclusions

In this study, the quality characteristics of 101 soybean sprouts were quantified. Moreover, the comprehensive evaluation of sprout quality characteristics was conducted using PCA and affiliation function analysis. The top 10 soybean materials exhibiting superior sprout characteristics were selected, which can be utilized to produce soybean sprouts or as parental materials in the breeding of new high-quality sprouts soybean varieties. In addition, this study proposed a mathematical model for the comprehensive evaluation of soybean sprout characteristics, suggesting that the sprout characteristics of soybeans could be comprehensively evaluated by five parameters: hypocotyl length, edible rate, 100-seed weight, and total isoflavone content and saponin content. The above findings not only provide excellent materials to produce soybean sprouts but also provide novel insights and a methodological foundation for the rapid and comprehensive evaluation of soybean sprout characteristics.

## Figures and Tables

**Table 1:** Statistical analysis of quality characteristics of soybean sprouts.

Indexes		Mean ± SD	Median	Minimum	Maximum	Range	CV (%)
Growth parameters	Hypocotyl length (cm)	7.56 ± 2.81	7.51	1.62	14.42	12.80	37.10
	Hypocotyl diameter (mm)	2.18 ± 0.26	2.10	1.70	3.00	1.30	12.00
	Edible rate (%)	88.14 ± 3.96	88.62	73.86	95.77	21.91	4.49
	100-seed weight (g)	14.02 ± 5.86	12.74	5.97	32.51	26.54	41.79
Bioactive components content	TPC (mgGAE g^−1^ DW)	2.71 ± 0.67	2.67	0.99	4.57	3.58	24.92
	TIC (μgGE g^−1^ DW)	10.48 ± 2.47	10.41	5.10	15.96	18.86	23.55
	Saponin content (mg g^−1^ DW)	63.24 ± 13.33	61.87	32.03	115.91	83.89	21.07
	GABA content (mg g^−1^ DW)	4.09 ± 1.59	4.00	0.28	9.10	8.82	38.73

**Table 2:** Principal components loading matrix and the eigenvalues of the quality characteristics of soybean sprouts.

Index	Principle Component		
	PC1	PC2	PC3
Hypocotyl length (cm)	−0.066	−0.414	0.437
Hypocotyl diameter (mm)	0.479	0.388	−0.023
Edible rate (%)	−0.336	0.472	0.344
100-seed weight (g)	−0.483	0.172	−0.397
TPC (mgGAE g^−1^)	0.487	0.371	0.004
TIC (μgGE g^−1^)	0.047	−0.207	0.564
Saponin content (mg g^−1^)	0.398	−0.442	−0.295
GABA content (mg g^−1^)	0.150	0.224	0.356
Eigen values	2.688	2.155	1.422
Contribution rate (%)	33.600	26.934	17.780
Cumulative contribution rate (%)	33.600	60.534	78.314

## Data Availability

The original contributions presented in the study are included in the article/Supplementary Material; further inquiries can be directed to the corresponding author.

## Supplementary Materials

The following supporting information can be downloaded at https://www.mdpi.com/article/10.3390/foods13213524/s1, Figure S1: The histogram of sprout quality characteristics; Table S1: The soybean materials used in this study; Table S2: The membership function values of 101 soybean sprouts; Table S3: The *D* value and rank of 101 soybean sprouts.
