# Supplementary material for: Diversity Analysis and Comprehensive Evaluation of 101 Soybean (Glycine max L.) Germplasms Based on Sprout Quality Characteristics
Source: Foods. 2024 Nov 4;13(21):3524. doi: 10.3390/foods13213524 (PMC11545536; doi:10.3390/foods13213524)
Supplement: Supplementary file 1 [file foods-13-03524-s001.zip › foods-3275377-supplementary.pdf]

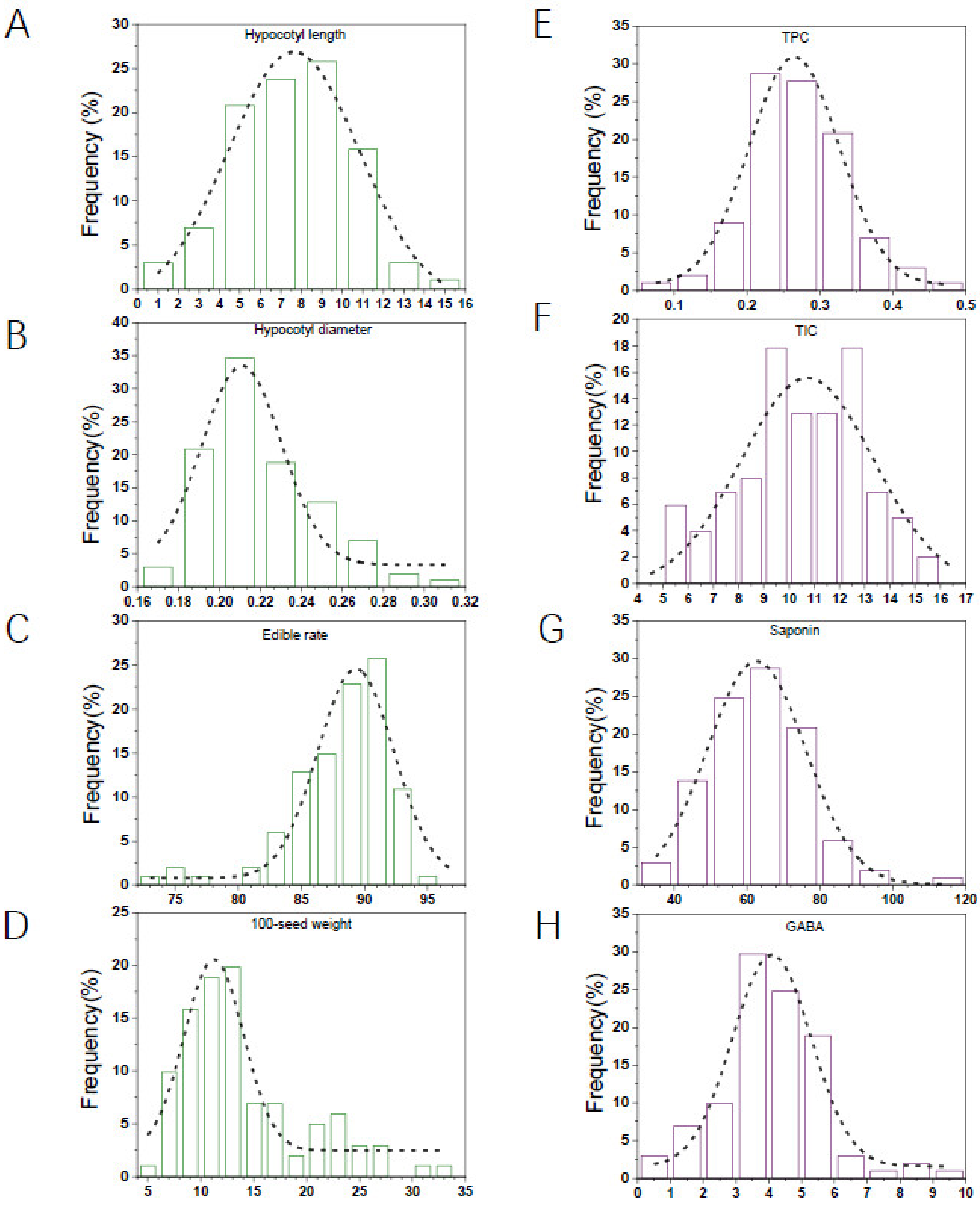

Figure S1. The histogram of sprouts quality characteristics

Table S1. The soybean materials used in this study.

Accession number	Accession name	Accession number	Accession name
**S1**	Taixing Liuyuexian	**S52**	Shuyang chundawuhuajia
**S2**	Huaiyin Wudou	**S53**	Huaiyin baihuaqiu
**S3**	Taizhou zihuawu	**S54**	Sheyang wandouhuangjia
**S4**	Taixing wudou	**S55**	Yancheng sancangzi
**S5**	Jiangyin wudou	**S56**	Dongtai zhongqiujiao
**S6**	Shuyang laobadou	**S57**	Dongtai bayuebajia
**S7**	Lianshui xiaobaihua	**S58**	Haian ciyudou1
**S8**	Lianshui xiaobaihua2	**S59**	Haian ciyudou2
**S9**	Huaiyin laobadou	**S60**	Haimen baiyouguozi
**S10**	Dongtai hongyoudou	**S61**	Qidong xianhaodou2
**S11**	Rugao zaorihong	**S62**	Qidong xiaoanhuangjia2
**S12**	Rugao zaojiahong2	**S63**	Wuxi wudou
**S13**	Qidong chundouB	**S64**	Rugao honghuadongdongqing1
**S14**	Nantong huangyouguozi	**S65**	Rugao honghuadongdongqing2
**S15**	Xinghua zaojiaguozi	**S66**	Rudong popifeng
**S16**	Fengxian xiaobaipi	**S67**	Rudong bayuebai
**S17**	Fengxian caohuangdou	**S68**	Haimen changshengguodou
**S18**	Fengxian daxihecaoA	**S69**	Nantong danhuangdouA
**S19**	Fengxian Daxi river coarse B	**S70**	Hanjiang hongdadou
**S20**	Peixian sanxiangjia	**S71**	Danyang wanchadou
**S21**	Peixian dabaijiao	**S72**	Liyang chadoubayuehuang
**S22**	Peixian dabaipi	**S73**	Taicang ziyuodou
**S23**	Tongshan baijiaqiao	**S74**	Suqian tuerdun1
**S24**	Tongshan xiaopingdinghuang	**S75**	Suqian tuerdun2
**S25**	Tongshan dalipingdinghuang	**S76**	Liyang xiangbianhuangdou
**S26**	Tongshan xiaopingdingwu	**S77**	Liyang xiangzidou
**S27**	Tongshan niumaohuang	**S78**	Jiangsu 73-13
**S28**	Tongshan liuxintianedan	**S79**	Rudong wanlvhuang1
**S29**	Tongshan zhengjidou	**S80**	Rudong wanlvhuang2

**S30**	Tongshan xihecao	**S81**	Haimen xifeng
**S31**	Tongshan hushandasili	**S82**	Yangzhou sanhuang
**S32**	Tongshan dajiandinghuang	**S83**	Zhenjiang huangdou
**S33**	Pixian baijianqiao	**S84**	Yixing qinghuangdou
**S34**	Pixian lianmaosheng	**S85**	Wujiang chishuzhong
**S35**	Pixian daerwo	**S86**	Taicang doufudou
**S36**	Pixian dawutiejia	**S87**	Rugao wanzhong
**S37**	Pixian layanghuang	**S88**	Tongshan xipi
**S38**	Xinyi xiaobaike	**S89**	Haimen huangqiao
**S39**	Xinyi xiaowuke	**S90**	Yangzhou wuyuedou
**S40**	Xinyi Yuenan dahuangke	**S91**	Shazhou chadou
**S41**	Xinyi big ocean iron angle	**S92**	Danyang huangzi
**S42**	Xinyi big white bark	**S93**	Dantu huangdou
**S43**	Xinyi small white bark	**S94**	Peixian hongmao
**S44**	Xinyi Tuhuan	**S95**	Peixian wuding
**S45**	Xinyi mud residue	**S96**	Lianshui baihuadou
**S46**	Xinyi beans	**S97**	Haimen baimaodou
**S47**	Xinyi flower face string beans	**S98**	Linhe xiangzidou
**S48**	Xinyi flower face string beans1	**S99**	Xinyi heikezi
**S49**	East China Sea pomegranate white flower rough	**S100**	Jindadou332
**S50**	East China Sea white flower rough	**S101**	chayuandou
**S51**	Shuyang red flower floor		

Table S2. The membership function values of 101 soybean sprouts

Code	U(X_1)	U(X_2)	U(X_3)	Code	U(X_1)	U(X_2)	U(X_3)
**S1**	1.272	1.509	1.167	**S52**	2.919	1.282	-0.083
**S2**	0.744	1.395	0.690	**S53**	2.933	1.423	0.466
**S3**	2.056	1.141	2.791	**S54**	2.602	1.477	1.849
**S4**	3.468	2.179	3.229	**S55**	2.722	1.484	0.534
**S5**	2.416	1.287	1.969	**S56**	1.972	0.706	0.685
**S6**	3.602	-0.374	0.324	**S57**	2.592	-0.150	1.532
**S7**	2.132	-0.595	1.755	**S58**	2.543	2.515	2.240
**S8**	2.455	-0.352	2.231	**S59**	1.981	1.335	2.046
**S9**	2.877	1.741	2.173	**S60**	2.030	0.659	2.423
**S10**	3.105	1.146	2.104	**S61**	2.766	0.883	1.447
**S11**	3.638	1.293	1.278	**S62**	2.639	0.485	0.884
**S12**	3.486	-0.589	1.675	**S63**	3.007	0.873	1.187
**S13**	2.775	0.499	2.646	**S64**	3.055	1.103	0.742
**S14**	2.639	0.507	1.889	**S65**	1.731	0.617	1.148
**S15**	3.180	1.048	1.727	**S66**	2.444	1.004	1.887
**S16**	3.308	1.704	1.704	**S67**	2.251	0.635	2.147
**S17**	2.903	-0.567	1.389	**S68**	1.704	0.828	2.585
**S18**	3.100	-0.281	0.584	**S69**	2.371	1.100	1.603
**S19**	3.525	1.393	1.971	**S70**	2.966	1.270	0.140
**S20**	2.134	1.161	2.170	**S71**	3.272	1.891	-0.275
**S21**	3.619	0.615	1.781	**S72**	1.940	2.439	2.409
**S22**	2.408	1.861	2.928	**S73**	1.937	1.026	2.058
**S23**	2.374	1.295	2.512	**S74**	2.281	0.607	0.337
**S24**	3.461	0.143	1.726	**S75**	2.396	3.048	3.521
**S25**	2.132	1.004	3.964	**S76**	1.995	0.745	0.454
**S26**	2.935	-0.216	1.111	**S77**	2.610	0.777	1.420
**S27**	2.720	-0.126	1.364	**S78**	1.154	0.680	0.011
**S28**	3.755	0.830	-0.016	**S79**	1.137	0.429	0.143
**S29**	3.055	0.889	1.946	**S80**	0.738	1.480	0.988
**S30**	3.148	0.889	2.408	**S81**	1.474	1.215	0.376
**S31**	3.222	0.630	2.681	**S82**	1.772	2.010	0.451
**S32**	3.952	-0.651	1.104	**S83**	0.316	0.794	1.515
**S33**	2.609	-0.686	0.964	**S84**	-0.131	0.286	0.915
**S34**	2.678	1.393	2.861	**S85**	0.872	1.477	1.482
**S35**	2.856	0.144	1.416	**S86**	1.435	1.421	0.120
**S36**	2.749	0.885	1.843	**S87**	2.333	1.234	0.135
**S37**	2.796	1.939	1.582	**S88**	3.216	2.969	1.079
**S38**	3.427	0.830	2.081	**S89**	2.585	2.394	0.555
**S39**	2.724	0.385	1.625	**S90**	2.502	2.326	-0.132
**S40**	2.319	1.063	2.722	**S91**	-0.923	-0.549	1.019
**S41**	3.144	0.904	2.193	**S92**	1.166	1.558	1.027

**S42**	3.444	1.448	1.150	**S93**	2.687	2.008	-1.000
**S43**	2.875	2.198	2.330	**S94**	2.182	2.303	0.576
**S44**	2.837	3.528	2.430	**S95**	3.072	3.784	0.577
**S45**	1.741	0.111	3.016	**S96**	2.863	2.831	-0.047
**S46**	2.207	1.082	2.231	**S97**	2.374	2.599	-0.332
**S47**	3.460	0.838	1.851	**S98**	3.402	2.853	1.256
**S48**	3.800	0.217	0.592	**S99**	3.646	2.901	1.170
**S49**	2.419	-1.000	1.995	**S100**	-1.000	1.873	1.023
**S50**	5.471	0.002	-0.087	**S101**	1.173	0.580	-0.516
**S51**	1.786	2.021	1.147				

Table S3. The *D* value and rank of 101 soybean sprouts

Accession number	*D* value	Rank	Accession number	*D* value	Rank
**S1**	1.330	81	**S52**	1.675	61
**S2**	0.955	94	**S53**	1.853	45
**S3**	1.908	39	**S54**	2.044	32
**S4**	2.971	2	**S55**	1.799	53
**S5**	1.926	37	**S56**	1.245	84
**S6**	1.490	72	**S57**	1.408	78
**S7**	1.109	91	**S58**	2.464	9
**S8**	1.439	75	**S59**	1.774	54
**S9**	2.327	14	**S60**	1.648	66
**S10**	2.204	22	**S61**	1.819	51
**S11**	2.296	15	**S62**	1.500	71
**S12**	1.673	62	**S63**	1.860	42
**S13**	1.963	36	**S64**	1.858	44
**S14**	1.736	57	**S65**	1.216	86
**S15**	2.117	29	**S66**	1.822	50
**S16**	2.392	11	**S67**	1.672	63
**S17**	1.366	80	**S68**	1.603	68
**S18**	1.366	79	**S69**	1.759	55
**S19**	2.439	10	**S70**	1.741	56
**S20**	1.808	52	**S71**	1.992	34
**S21**	2.168	26	**S72**	2.218	20
**S22**	2.338	12	**S73**	1.651	65
**S23**	2.034	33	**S74**	1.264	83
**S24**	1.926	38	**S75**	2.875	3
**S25**	2.160	27	**S76**	1.215	87
**S26**	1.437	76	**S77**	1.709	60
**S27**	1.433	77	**S78**	0.731	96

**S28**	1.893	41	**S79**	0.668	97
**S29**	2.059	31	**S80**	1.050	93
**S30**	2.203	23	**S81**	1.135	89
**S31**	2.207	21	**S82**	1.554	70
**S32**	1.722	58	**S83**	0.753	95
**S33**	1.102	92	**S84**	0.250	100
**S34**	2.278	16	**S85**	1.219	85
**S35**	1.596	69	**S86**	1.131	90
**S36**	1.902	40	**S87**	1.456	74
**S37**	2.226	19	**S88**	2.646	7
**S38**	2.228	18	**S89**	2.059	30
**S39**	1.670	64	**S90**	1.844	46
**S40**	1.979	35	**S91**	-0.353	101
**S41**	2.158	28	**S92**	1.269	82
**S42**	2.236	17	**S93**	1.616	67
**S43**	2.519	8	**S94**	1.859	43
**S44**	2.982	1	**S95**	2.750	5
**S45**	1.470	73	**S96**	2.192	25
**S46**	1.826	49	**S97**	1.837	48
**S47**	2.193	24	**S98**	2.726	6
**S48**	1.840	47	**S99**	2.828	4
**S49**	1.147	88	**S100**	0.447	99
**S50**	2.328	13	**S101**	0.586	98
**S51**	1.722	59			
